# Integrating graphene oxide into layers of PVDF/PVDF@cross-linked sodium alginate/polyamide membrane for efficiently enhancing desalination performances

**DOI:** 10.1038/s41598-022-21316-y

**Published:** 2022-10-07

**Authors:** Zohreh Mohammadi, Mir Saeed Seyed Dorraji, Arsalan Ahmadi, Abdolreza Tarighati Sareshkeh, Mohammad Hossein Rasoulifard

**Affiliations:** 1 Applied Chemistry Research Laboratory, Department of Chemistry, Faculty of Science, University of Zanjan, Zanjan, Iran; 2 Research and Development Laboratory, Absamin Water Treatment Co., Karaj, Iran

**Keywords:** Chemistry, Engineering, Materials science

## Abstract

The membrane modules of the water treatment system are faced costly damages; thereby executing pre-desalination units based on Nanofiltration (NF) could prevent these suffers, and improve the permeated water flux (PWF) and salt rejection (SR). Hence, we focused on the construction of a novel ternary-layer NF membrane through “electrospinning Polyvinylidene Fluoride (PVDF) (as bottom layer)”, “generating middle layer by electrospinning PVDF along with, the implementation cross-linking after electrospraying Sodium Alginate”, and “synthesizing Polyamide (as top layer) through interfacial polymerization”. More importantly, it anticipated that the Taguchi statistical method can expeditiously optimize the effects of Graphene Oxide nano-sheets (GOns) on water-dependent properties, such as PWF and SR. Astonishingly, the desalination capabilities significantly improved, when the top, middle, and bottom layers simultaneously had 1, 0.1, and 0.1 wt.% of GOns, respectively. Overall, comparing the performances between the optimized sample containing low-dosage and without GOns demonstrated the PWF ameliorated from 6.68 to 20.36 L/m^2^ h; also, the SR ability remained on an incremental basis as NaCl < MgCl_2_ < MgSO_4_ under 6 bar pressure. Manifestly, these authentic results denoted promising, innovative, and large-scaling insights when effectual PWF and SR be necessary.

## Introduction

Nowadays, the human population and industries are growing dramatically, demonstrating that the need for high-quality water is an urgently issue^1^. By considering the type of contaminants, different polluted-water remediation methods define to eliminate various contaminants from water, such as Filtration^2^, Electrochemical^3^, Adsorption^4^, and Advanced Oxidation Process^5^. The desalination of surface waters is the reliable separation technology via the ion-sensitive membranes, which subsequently suggests that the accessibility to chemical precursors and the economic strategies are indispensable challenges in preparing NF membranes. Therefore, the physicochemical properties of membranes should be amend using different polymeric materials and non-zero-charged nanoparticles^6^.

Lately, NF membrane has attracted much attention for employment in a specific water purification technology, such as wastewater treatment, distillation, pollutants adsorption, and bio-products recovery. Therefore, most researchers attempt to utilize NF membrane owing to outstanding advantages, including the practical rejection of ions or low molecular weight organic materials (approximately ranging from 200 to 1000 g/mol), the spectacular of the PWF, the lower hydraulic pressure than Reverse Osmosis, and the inexpensive process by energy-saving strategies^7,8^.

In recent years, a popular method to produce NF membranes is electrospinning due to the production of non-woven nano-fibrous along with the pore size distribution in the micrometer range. To that end, some unique features could endow to high porosity (> 80%) NF membrane, including numerous interconnected pores, great flexibility, high specific surface area, good liquid permeability, and perfect capability for chemical manipulations^9^. One of the most famous polymers to fabricate a harmonic NF membrane is PVDF that has exciting properties, viz. superb electro-spinnability, excellent thermal stability, and extraordinary physicochemical durability against a wide range of solvents, pHs, and tensions. Therefore, these intrinsic benefits generally caused PVDF considered as a tenacious support layer in constructing multi-layer membranes. Nevertheless, PVDF has an intrinsic hydrophobicity nature that needs to be composite with other additives to increase PWF. Besides adding hydrophilic materials, generating a thin film layer (TFL) on the electrospun nanofibers of PVDF through Layer-By-Layer (LBL) technique is a facile way to create an asymmetric NF membrane with acceptable PWF and admirable SR^10,11^.

Hitherto, an eco-friendly candidate to prepare an asymmetric NF membrane could be the natural-based polysaccharides or their manipulated derivatives. For instance, SA obtains from various resources such as microorganisms or brown algae, which have some benefits in the membrane, including negatively charged structure, non-toxic, and the high capability of film-forming. A safe process for gelling SA performs by cross-linking polymeric chains with multivalent cations that effectively restrict unfavorable water solubility of SA^12,13^. Furthermore, the ionic coordination occurs between the negatively charged carboxyl or hydroxyl sites of SA chains and Ca^2+^ ions (an eco-friendly cross-linking agent). In the cross-linked state of SA (CSA), most hydrophilic functional groups get involved in cross-linking polymeric chains of alginate, resulting in water molecules' difficulty penetrating CSA in the closer alginate chains of CSA compared to non-cross-linked of SA. Thus, the cross-linked state of SA controls the swelling degree of alginate hydrogel; however, it causes to reveal some conspicuous drawbacks, such as dropping PWF^14^.

In addition to preparing CSA, a hydrophobic and salt-sensitive layer could fabricate by conventional interfacial polymerization of tri-Mesoyl Chloride (TMC) and Polyamine monomers, rendering create a PA layer, suggesting that the TFL of PA could utilize in NF membrane. The performances of PA depend on the used monomers for preparing PA and the operational conditions (e.g., the pump pressure, and the type and concentration of salt). Overall, PA has excellent SR and chemical stability; hence, the used precursors and synthesizing method could donate special features to the PA layer in thin-film composite (TFC) NF membrane^1,15–17^.

An abundant, low-cost, and traditional nanostructure is GOns that has many aspirants for preparing nanocomposites in different fields^18^. Some prominent features make to employing GOns in membranes are the vast lamellar structure, the excellent mechanical properties, the negatively charged nature, the hydrophobic functional groups, the ability to create hydrogen bonding with various polymers, the high capability for complexing with multivalent ions, the suitable dispersibility at aprotic solvents and, the nano-channels and nano-pores for penetrating water molecules. Therefore, GOns significantly endow high water permeability, excellent anions exclusion, and good mechanical properties to our NF membrane^19,20^.

The statistical experimental design methods assess the effects of interactions among the operating parameters (i.e., factors). When the Taguchi method adopts and applies mathematical and statistical techniques, this approach could become attractive to save money and time by decreasing the number of required experiments. Therefore, the designed arrays in Taguchi software cause optimizing the factors, studying the influence of each parameter, revealing the correlations among factors, and permitting the parameters to have the greatest or optimal level. Moreover, the Analysis of Variance (ANOVA) usually provides correct information about factors. In addition, analyzing the obtained experimental results are statistically revealed identifying the portion of factors, and establishing the relationships between factors^21–23^.

As known, the membrane setups for desalination always exposes to attacking high-concentration salts; thereby, they absolutely preserve by inexpensive pre-treatment units. Therefore, the primary duty of these units universally is to decrease the negative impacts of salts on the main setups, consequently results in the pre-desalination unit must effectively diminish salt concentration. In this research, we decided to construct a NF membrane with the help of TFC technology for execution as a pre-desalination unit, thereby it focused on the selection of abundant and low-cost precursors; also, accomplishing simple processes toward the preparation of the ternary-layers membrane that is able to have reasonable SR and PWF. Furthermore, the effect of GOns on PWF and SR evaluated by the Taguchi method to certify the superiority of the TFC NF membrane. Optimistically, GOns could barely increase wettability and negatively charge towards achieving desirable PWF and SR, respectively. Yet, no similar work has been reported about the GOns-composited PC_1.5_PP for desalination by the addressed methods.

## Reagents and experimental

### Materials

GOns (99.5%, 2–18 nm thickness) purchased from U.S. Research Nanomaterials Co., (USA). PVDF powder (Molecular weight: 370,000 g.mol^−1^) and tri-Mesoyl Chloride (TMC) were bought from Sigma-Aldrich. Dimethylformamide (DMF), Acetone, n-Hexane, tri-Ethylamine (TEA), Calcium Chloride dihydrate (CaCl_2_.2H_2_O), Magnesium Chloride anhydrous (MgCl_2_), Magnesium Sulfate anhydrous (MgSO_4_), and Sodium Chloride (NaCl) supplied from Merck Co., (Germany). Sodium Alginate (SA) powder received from Alpha-aesar Company. Meta-Phenylenediamine (MPD) obtained from Exir Company.

### Fabrication of samples

#### Preparation of PVDF//SA_x_@PVDF (PS_x_P)

8 gr PVDF powder was dissolved in 92 g DMF/Acetone mixture (with a volume ratio of 2 to 3) and stirred for 24 h (Solution A). Moreover, 0.0125, 0.075, or 0.15 g SA dissolved in 5 mL deionized water to prepare the SA_x_ section in the SA_x_@PVDF layer at room temperature, where x assign to 0.25, 1.5, or 3 wt.% of SA, respectively (Solution B). Simultaneously, the solutions of A and B were spun and sprayed via two nuzzles for 1.5 h, respectively. Then, the remained of solution A spun until a pure PVDF layer created over the SA_x_@PVDF layer (precisely i.e., the PSxP was produced). The electrospinning conditions reported in Table S1.

#### Fabricating PVDF//CSA_x_@PVDF (PC_x_P)

Firstly, the fabricated samples of PS_x_P immersed in deionized water for maximum overspreading of SA for 0.5 h. Then, 0.37, 1.47, or 2.94 g CaCl_2_.2H_2_O dissolved in 100 ml deionized water for preparing the solutions of 0.025, 0.1, and 0.2 mol L^−1^ CaCl_2_, respectively. Secondary, the as-prepared samples of PS_0.25_P, PS_1.5_P, or PS_3_P immersed in the cross-linking solution of CaCl_2_ (i.e., 0.025, 0.1, or 0.2 mol L^−1^, respectively) for six hours at room temperature, which subsequently results in PC_0.25_P, PC_1.5_P or PC_3_P samples obtained, respectively. Finally, the PC_x_P samples rinsed with deionized water to remove the unreacted reagents.

#### The synthesis of PA layer over PC_1.5_P (PVDF//CSA_x_@PVDF//PA (PC_1.5_PP))

Firstly, 0.512 g MPD dissolved in 25 mL deionized water; then, 1 ml of 2 wt.% TEA solution (as a catalyst) injected into this solution. The sample of PC_1.5_P immersed in MPD solution for 5 min; subsequently, the excess amount of MPD monomers drained off by a plastic rod. On another dish, 0.024 g TMC dissolved in 25 mL n-Hexane; after, the impregnated amine of PC_1.5_P soaked in this solution for 45 s towards the interfacial polymerization of PA TFL over PC_1.5_P. Finally, to accomplish the polymerization process of PA, the obtained PC_1.5_PP sample cured in an oven at 80 ^◦^C for 10 min.

#### The preparation of PC_1.5_PP membrane composited with GOns as TFC NF membrane

**Figure 1:**
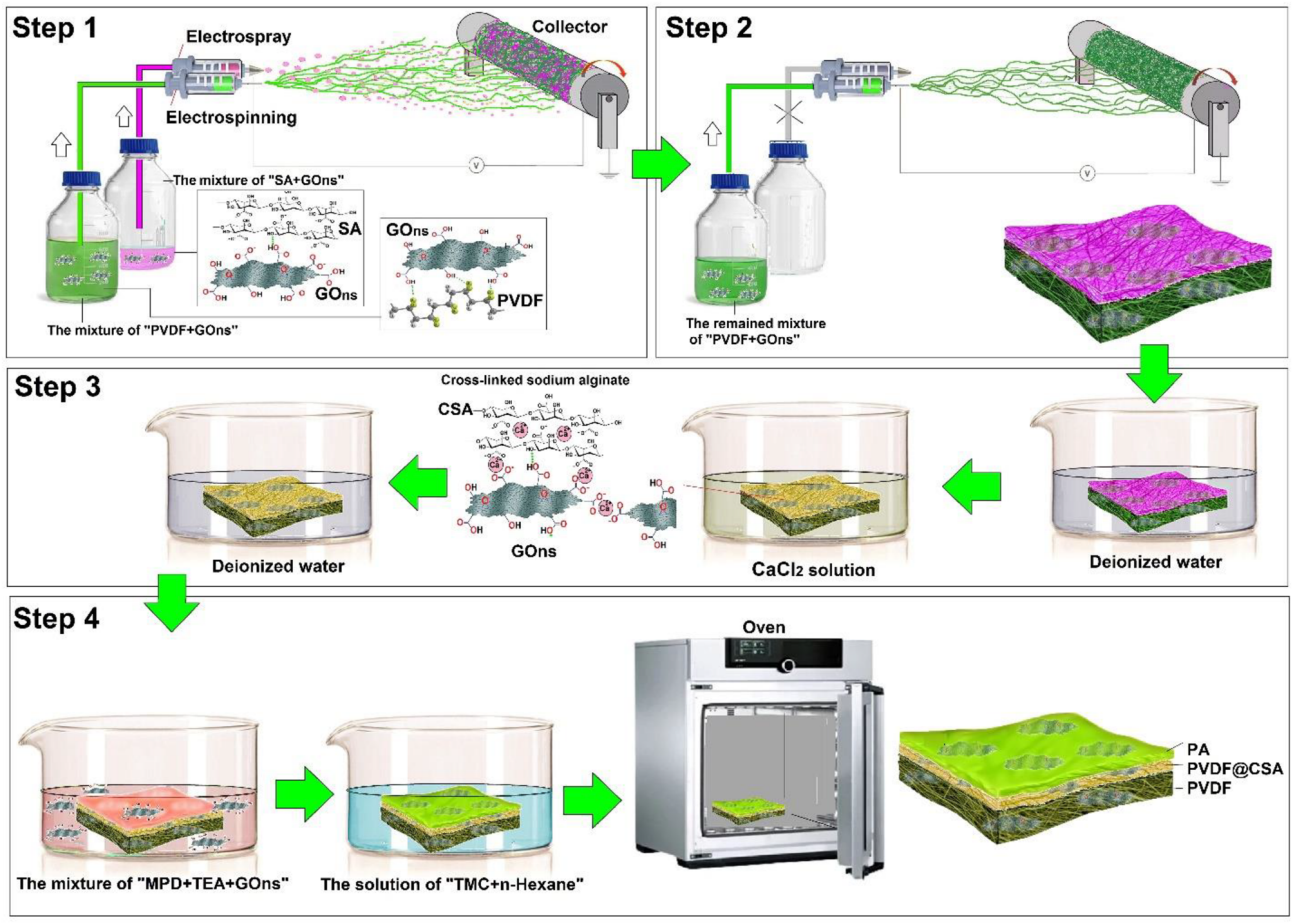
Schematic of the preparation of PC_1.5_PP TFC NF membrane (Step1: primary synthesizing the middle layer by electrospinning and electrospraying techniques. Step2: The complete preparation of the bottom layer by electrospinning. Step3: Crosslinking SA by immersion method to complete preparation of the middle layer. Step4: The construction of PA TFL by interfacial polymerization method).

GOns could have an undeniable role in increasing PWF and SR performances; subsequently, the different amounts of GOns could add to PVDF, SA, and MPD solutions (Tables S2, S3, and Fig. 1).

I. For preparing “PVDF + GOns”, 0.8, 4, or 8 g GOns dispersed in 92 g the mixture of DMF/Acetone by ultrasound for 30 min. Then, 8 g PVDF powder dissolved in this suspension for 24 h.
II. For preparing “SA + GOns”, 0.001, 0.007, or 0.015 g GOns mixed in 100 mL deionized water; subsequently, it sonicated for 20 min. Then, 0.152 g SA powder dissolved in this suspension for 24 h.
III. For preparing “MPD + GOns”, 0.005, 0.025, or 0.05 g GOns sonicated into 200 mL deionized water for 20 min; then, 5.12 g MPD and 1 ml of 2 wt.% TEA solution added to this suspension.

According to the reported information in Tables S2 and S3, these mixtures used instead of their primary solutions. Furthermore, QUALITEK-4 software employed to design experiments based on the Taguchi method. Likewise, the introduced amounts of GOns to the software attributed to weight percentage.

### The SR and the PWF assessment

A laboratory-built dead-end stirred system designed to evaluate the performances of samples in the salt separation process from water. In this cell, the accessible surface area of the membrane equaled 12.57 cm^2^. Before performing the permeation tests, the membrane was compacted to attain the steady state of PWF under seven bar for 0.5 h. Then, in the operating pressure of six bar, the PWF calculated by measuring the permeated water every 10 min for one hour. The used equation for calculating PWF is as follows (Eq. 1), where V, A, and Δt are the volume of permeated-water (L), the effective surface area of membrane (m^2^), and filtration time (h), respectively^7^:

1 $${\text{PWF}} = \frac{{\text{V}}}{{{\text{A}} \times \Delta {\text{t}}}}$$

The feed contains 2000 mg L^−1^ of salt (e.g., NaCl, MgCl_2_, and MgSO_4_). The percentage of SR calculated by Eq. 2 at room temperature. The feed solution and the permeated water have particular electrical conductivity values. C_f_ and C_p_ are the electrical conductivity of the feed solution and the permeated water, respectively^8^.

2 $${\text{SR}} = \frac{{{\text{C}}_{{\text{f}}} - {\text{C}}_{{\text{p}}} }}{{{\text{C}}_{{\text{f}}} }} \times 100$$

### Evaluation of water uptake capacity

The physicochemical nature of the membrane to absorb water depends on the type and number of hydrophilic functional groups on polymeric chains. Therefore, the control of the swelling degree of the NF membrane should decrease to obtain higher water-dependent performances. The area of the membrane sample (3 $\times$ 3 cm^2^) cut, dried, and weighted (m_D_); then, it immersed in a CaCl_2_ bath for one hour, and the sample was rinsed with deionized water. The excess moisture taken through contact with ordinary filter paper. Then, it was weighted (m_S_) at room temperature. Eventually, the percentage of water uptake calculated by Eq. 3^13^.

3 $${\text{Water}}\;{\text{Uptake}} = \frac{{{\text{m}}_{{\text{S}}} - {\text{m}}_{{\text{D}}} }}{{{\text{m}}_{{\text{D}}} }} \times 100$$

### Characterization

The used electrospinning device (Model ES1000, Fanavaran Nanomeghyas Co., Iran) employed for electrospinning and electrospraying. Fourier Transform Infrared spectroscopy (FTIR; Perkin-Elmer, USA) used to analyze the type of GOns interactions in each layer. The morphology of layers characterized by Scanning Electron Microscopy (SEM; Philips, CM10, Dutch). The hydrophobicity measured via Water Contact Angle (WCA) value (with the help of a Handheld Digital Microscope, Dino lite AM2111 model, Taiwan). Then, ImageJ software applied to calculate the WCA value. The pore size distribution of samples investigated by scanning the SEM images in the ImageJ software, the Excel software executed for analyzing the extracted data. The conductivity of water samples revealed by a conductometer (Milwaukee MW01 PRO, Milwaukee, USA).

## Results and discussion

### Studying the prepared samples of PS_x_P and PC_x_P

**Figure 2:**
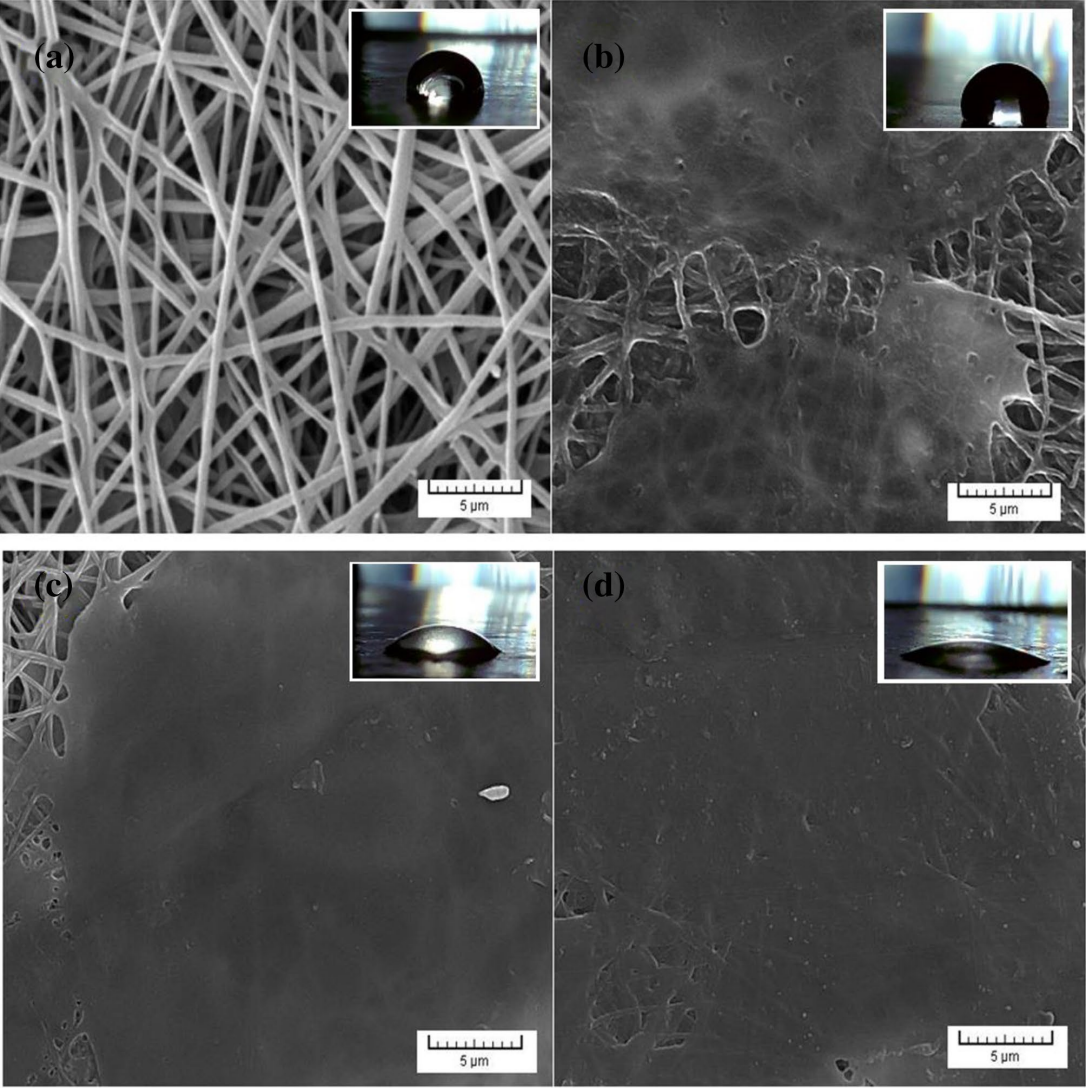
SEM images of (**a**) PVDF nano-fibrous, (**b**) PS_0.25_P, (**c**) PS_1.5_P, and (**d**) PS_3_P, which WCA values are (**a**) 140.66°, (**b**) 109.6°, (**c**) 47.02°, and (**d**) 27.24°, respectively.

Firstly, the measured thickness of PVDF, PS_0.25_P, PS_1.5_P, and PS_3_P samples were 33, 35, 36, and 38 µm after the drying process, respectively. As obviously displayed in Fig. 2a, PVDF nano-fibrous have a uniform scaffold, which means the electrospinning process of PVDF performed without any shortcomings. Besides, Fig. 2b, c demonstrates that increasing SA concentration led to more uniformly flattened SA. In the PS_3_P sample (Fig. 2d), the excessive concentration of SA retracts the sprayed drop of SA due to increased hydrogen bonding of SA chains. It creates an extensive three-dimensional network and less mobility of polymeric chains compared to when the used concentration of SA was 0.25 or 1.5 wt.%. On the other hand, the high concentration of SA could enhance wettability owing to SA has the hydrophilic nature that is suitable for creating hydrogen bonding with water molecules. Therefore, the SA concentration of up to 3 wt.% led to decreasing WCA from 140.66° to 27.24° in PVDF and PS_3_P samples, respectively^1,7,24–28^.

**Figure 3:**
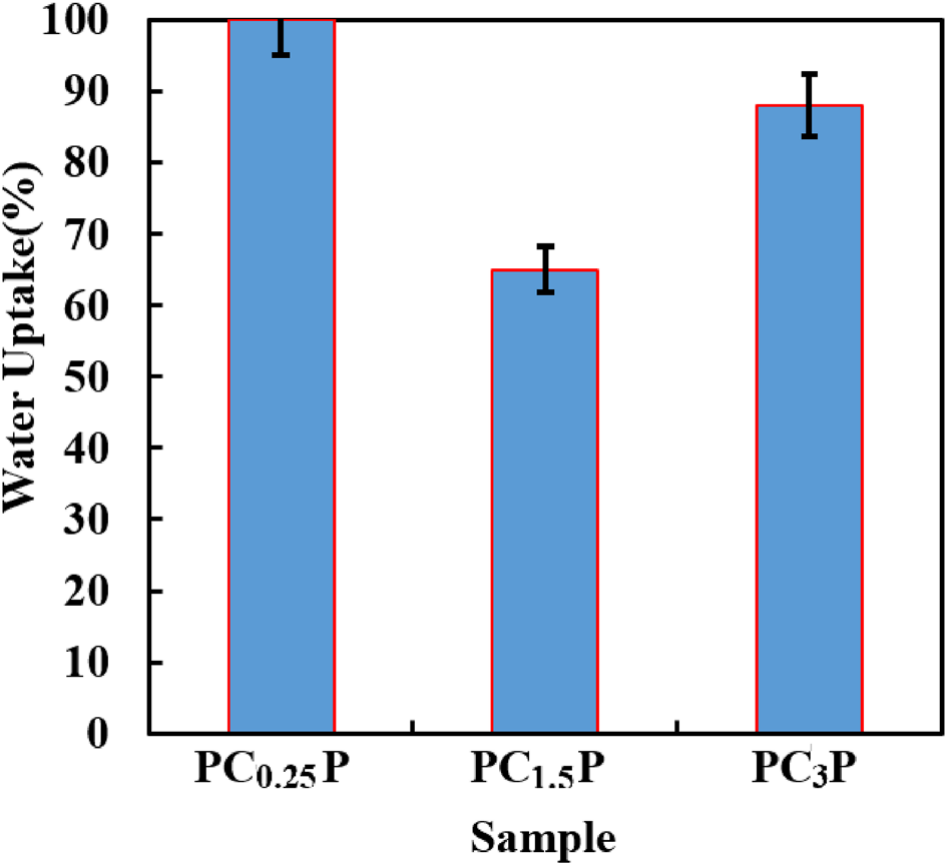
Comparison of water uptake in PC_x_P samples.

Water uptake is a critical aspect of wettability and cross-linking density, which originates from the chemical composition of the membrane. Ca^2+^ ion, as the cross-linking agent of SA, makes to create a three-dimension network of CSA. In other words, Guluronate unites of SA cross-linked together as a homo-polymeric block at the presence of Ca^2+^ (Fig. S1). The obtained hydrogel called “egg-box”, which has a closer distance between alginate chains than SA. Therefore, the higher cross-linked density of chains limits water penetration in hydrogel due to closer alginate chains^29,30^. It led to the less value of water uptake in the PC_1.5_P sample (Fig. 3), which could be due to the maximum cross-linking density that diminishes holding water into alginate hydrogel. In PC_0.25_P and PC_3_P samples, the egg-box junctions could generate high discontinuity between alginate chains^5,13,31^. In addition to these, as depicted in Fig. S2, the lowest PWF attributed to the PC_1.5_P sample, originating from the truth that the maximum cross-linking density of CSA and the less swelling degree of CSA could occur in this sample; hence, this sample was selected as the best sample for construction PC_1.5_PP membrane^13^.

**Figure 4:**
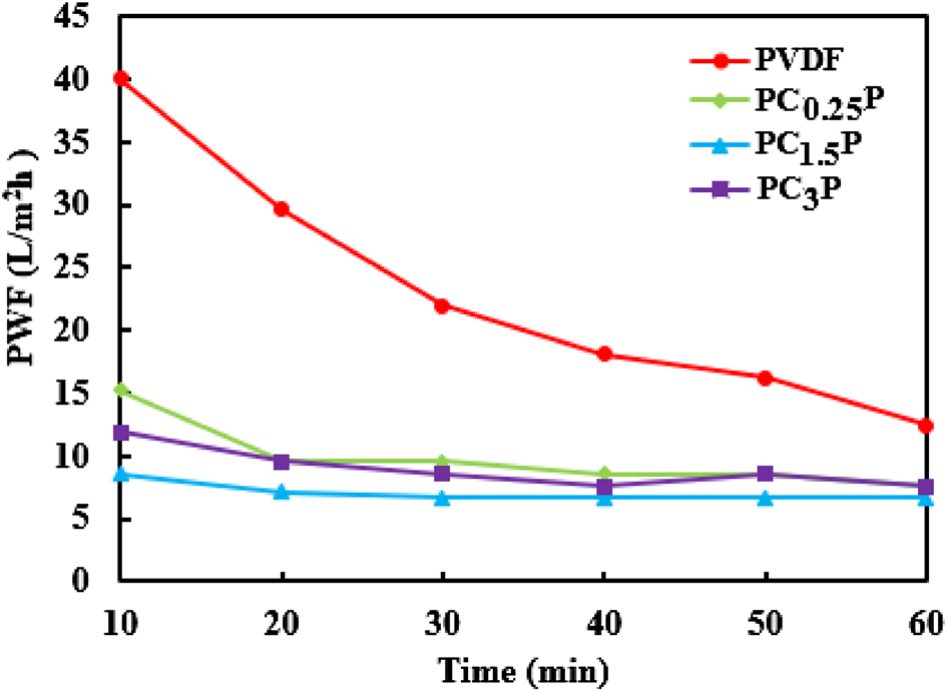
The effect of CSA concentration on PWF.

As depicted in Fig. 4, the PWF of PC_x_P samples is lower than PVDF nano-fibrous because CSA effectively diminished the accessible pores on PVDF nano-fibrous and restricted penetrating water molecules on PVDF nano-fibrous. A simple comparison between PC_0.25_P and PC_3_P samples demonstrated that the insufficient cross-linking density led to higher hydrophilicity and more WCA than PC_1.5_P. Therefore, most water molecules could trap between uncross-linked alginate chains instead of passing from these samples. In addition, the lowest PWF belonged to PC_1.5_P, which is related to the high cross-linking density, low swelling degree, uniform dispersion of alginate, and sufficient hydrophilicity^14,16^.

### Surveying the effect of GOns on PC_1.5_PP membrane

**Figure 5:**
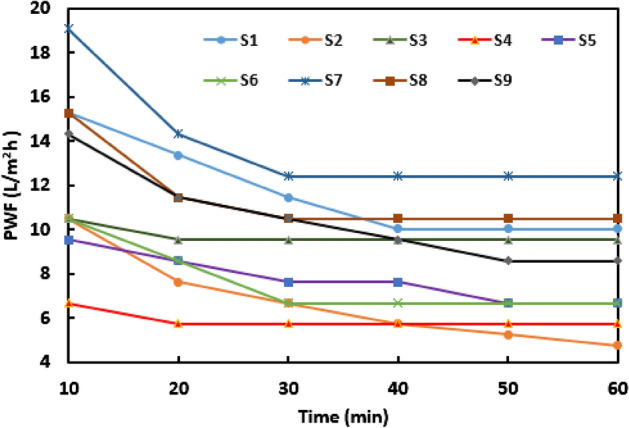
The effect of GOns on PWF of the prepared NF samples.

**Figure 6:**
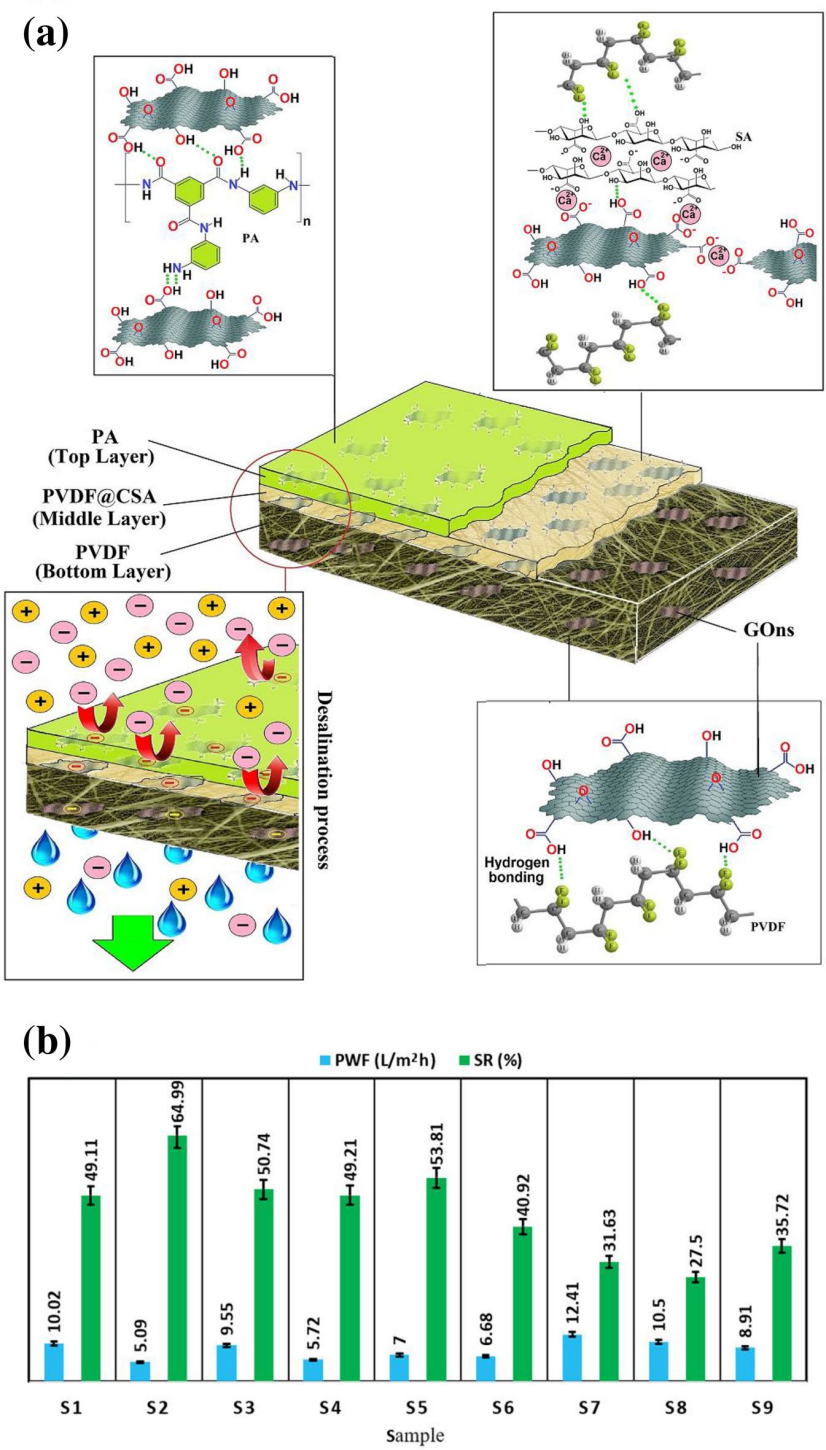
(**a**) The final schematic of the GOns-modified membrane, and (**b**) the effect of GOns on PWF and SR of samples from S1 to S9 samples.

The addition of GOns (as one of the hydrophilic structures) to the membrane could increase PWF and SR. Indeed, the faster water molecules penetration arises from creating nano-channels between polymeric chains and edges’ GOns, and the nano-pores of GOns surface. Besides, negatively charged GOns could effectively sensitize the membrane against penetrating anions through the Donnan exclusion effect. According to Table S3 and Fig. 5, the prepared NF samples made by the LBL technique. As the obtained results of the samples from S1 to S9, the PWF slowly dropped, and all of them reached a steady trend after a while. The main reason for these initial downtrends presumably attributed to compacting polymeric chains and decreasing the size of void spaces by operational pressure. A comparative overview to Fig. 5 ends in Fig. 6, which clearly illustrates that the maximum PWF and SR belong to S7 (12.41 L/m^2^ h) and S2 (65%), respectively. Meanwhile, the minimum amounts of PWF and SR obtained in samples of S2 (5.09 L/m^2^ h) and S8 (27.5%), respectively^32–34^. Overall, when the cross-linking density of middle layer controlled and the desired amount of GOns added in each layer of PC_1.5_PP, the samples of S1-S9 revealed unique desalination performances. These revealed that GOns dosage had crucial effect on the physicochemical properties of membrane, SR, and PWF^13^.

In order to find the best TFC NF sample with the highest performances, the effectiveness of each parameter could survey via ANOVA. For this purpose, the used Taguchi method can extract critical information. Meanwhile, the without-dimension comparable responses could calculate by the Overall Evaluation Criteria (OEC) (Eq. 4). According to this equation, the effective individual criteria of “Y_i_, Y_j_, and Y_k_”, “W_i_, W_j_, and W_k_”, “Y_is_, Y_js_, and Y_ks_” and “Y_ib_, Y_jb_, and Y_kb_” respectively belong to final responses, relative weight, worst value, and best value. Therefore, the PWF and SR (as the performances of TFC NF nanocomposite) have the same response weight (50%) for characterizing the effect of GOns in each layer. Furthermore, the quality characteristic of responses is based on “the bigger value is better”, and the obtained ANOVA results from Eq. 4 are exposure in Table S4^1,21–23^.

4 $$OEC = \left( {\left[ {\frac{{Y_{i} - Y_{is} }}{{Y_{ib} - Y_{is} }}} \right] \times W_{i} } \right) + \left( {\left[ {1 - \frac{{\left| {Y_{j} - Y_{js} } \right|}}{{Y_{ib} - Y_{is} }}} \right] \times W_{j} } \right) + \left( {\left[ {1 - \frac{{\left| {Y_{k} - Y_{ks} } \right|}}{{Y_{ib} - Y_{is} }}} \right] \times W_{k} } \right)$$

**Figure 7:**
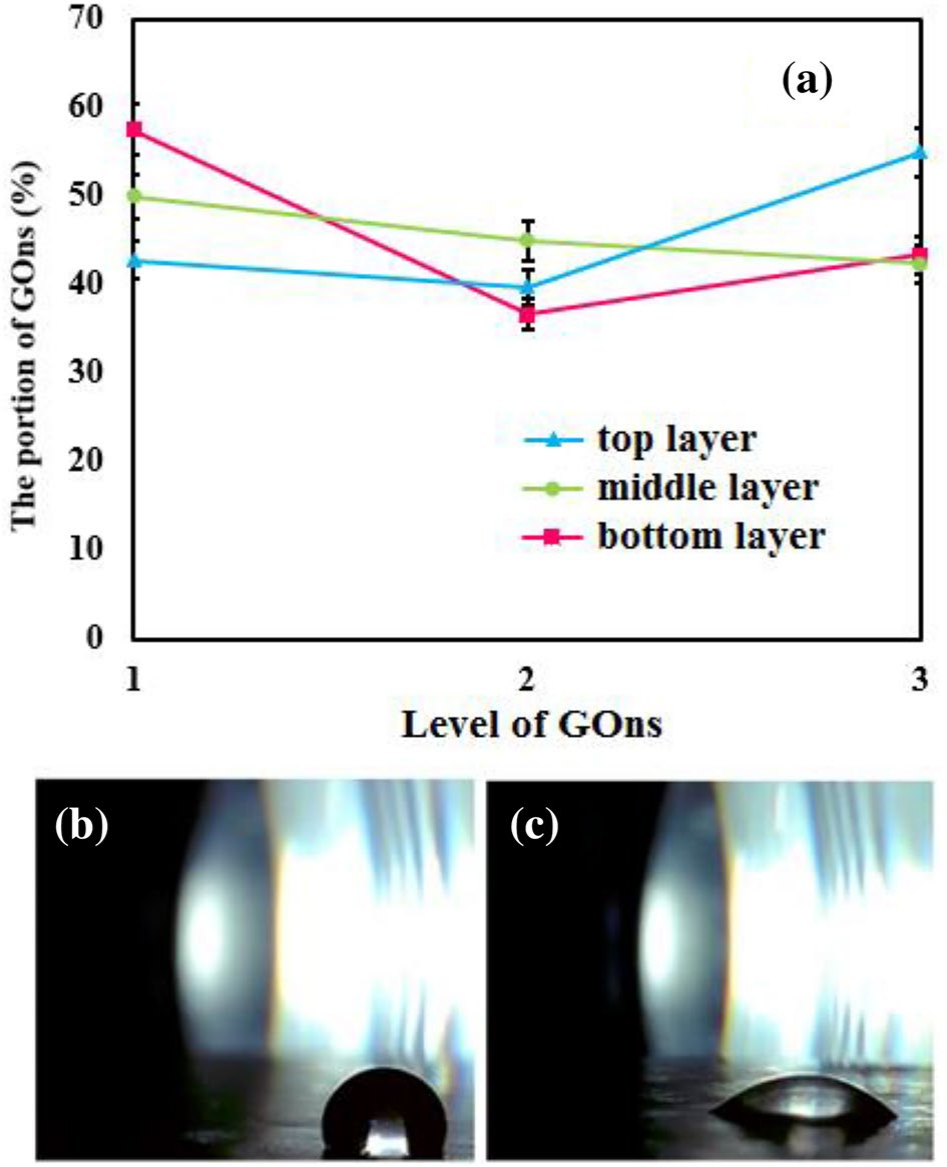
(**a**) Response diagram of the effectiveness of GOns levels on the PWF of PC_1.5_PP nanocomposite. WCA of samples: (**b**) 0.1 wt.% GOns in PVDF (119.26°) and (**c**) the optimized amounts of GOns in each layer of PC_1.5_PP (40.07°).

The optimization of effective parameters performed through ANOVA and displayed in Fig. 7a. The best PWF revealed, when the levels of GOns were G1 = 3, G2 = 1, and G3 = 1 for the layer of top, middle, and bottom, respectively. Therefore, PWF effectively increases, when top, middle, and bottom layers simultaneously have 1, 0.1, and 0.1 wt.% of GOns, respectively. The hydrophilic property of PA and GOns synergistically causes to the penetration of water molecules in the PC_1.5_PP sample. Thus, the PWF equaled 20.36 L/m^2^.h, when each of the three layers was composed with the proper amounts of GOns. While, the PWF obtained 6.68 L/m^2^.h, when the PC_1.5_PP sample was empty of GOns. As shown in Fig. S3, the higher amounts of GOns in the middle and bottom layers led to the remarkable aggregation of GOns. Eventually, the attained data from ANOVA revealed that the optimized-amount portion of GOns in top, middle, and bottom layers are 57.5%, 10.59%, and 22.32%, respectively^34–37^. Moreover, the presence of GOns in PC_1.5_P caused to diminish the WCA value than pure PVDF nano-fibrous (140.66°) (Fig. 7b, c). Therefore, the GOns-composed PC_1.5_PP membrane exhibited the lowest WCA and the highest PWF, attributing to the hydrophilic nature of GOns that can effectively dominate on the hydrophobic nature of PVDF and CSA^38,39^.

**Figure 8:**
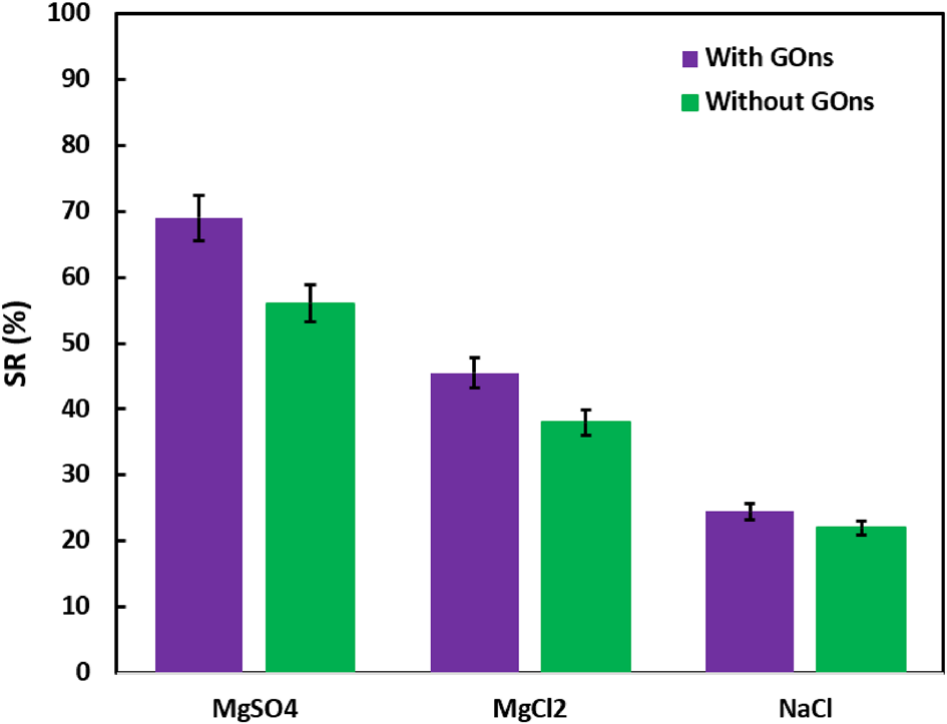
The SR in PC_1.5_PP samples.

The effect of size exclusion and Donnan exclusion characterized by a 2000 mg L^−1^ solution of NaCl, MgCl_2_, or MgSO_4_ in the GOns-integrated PC_1.5_PP membrane. As displayed in Fig. 8, the yields of SR were 24.5, 45.4, and 69.3% for NaCl, MgCl_2_, and MgSO_4_, respectively. The achieved results return to the more ion radius and the higher charge density of SO_4_^2−^ than Cl^−^, which leads to lower penetration of SO_4_^2−^ into the membrane. Moreover, penetrating rate of NaCl is higher compared to MgCl_2_ that is owing to the facts that (I) the diffusion coefficient of NaCl (1.48 × 10^–9^ m^2^ s^−1^) is more than MgCl_2_ (1.04 × 10^–9^ m^2^ s^−1^), (II) the hydrated ionic diameter of Cl^−^ (0.66 nm), Na^+^ (0.72 nm), SO_4_^2−^ (0.76 nm) and Mg^2+^ (0.86 nm). Therefore, the bigger size of ions (i.e., multivalent ions) difficulty penetrate on the membrane. Thus, the SR of NaCl < MgCl_2_ < MgSO_4_ could be explained by all these justifications^40,41^.

GOns have promising potential for preparing high-performance nanocomposite membranes, which could overcome the intrinsic shortcomings of polymeric matrixes. The main reason for this choice could attribute to the chemical interactions between oxygen-based functional groups of GOns and amine groups of MPD or TMC, which effectively could lead to cross-link PA chains, increase hydrophilicity, improve mechanical durability, higher PWF, and enhance the negative charge in membrane^42^. As demonstrated in Fig. S4a, the peaks at around 1180, 1650 nm^−1^, and 2900 nm^−1^ assign to the stretching vibration of –C(F_2_)–, the absorption of C–C, and the stretching vibrations of C-H, respectively. As Fig. S4b, the absorption peaks of GOns from 2850 to 3020 nm^−1^ attribute to the asymmetric or symmetric vibrations of C-H, and the broad peak at around 3400 nm^−1^ corresponds to the stretching vibrations of –OH. Besides, the peaks at 1070, 1240, 1400, and 1750 nm^−1^ correspond to the stretching vibrations of C–O, C–O–C, phenolic carboxylic acid, and C=O, respectively^43–45^. As Fig. S4c, d, SA is a polysaccharide with the polar functional groups, such as –OH and –COONa. In addition to the broad peak of –OH, the symmetric or asymmetric stretching vibrations of carboxylate were detected at around 1580 and 1070 nm^−1^. The process of cross-linking SA via Ca^2+^ led to creation of a three-dimensional network in the hydrogel of alginate, resulting in the alginate peaks shifted from 1414 to 1420 nm^−1^^46–48^. Fig. S4e contains all of the peaks, which attributed to PVDF, GOns, and CSA. Furthermore, the peaks at around 1550, 1620, and 1680 nm^−1^ belong to the unreacted N–H (amine state), reacted N–H (amide state), and C=O (amide state), respectively. Likewise, the low intensity of the –OH peak corresponds to cross-linked GOns with PA. Thus, the PC_1.5_PP nanocomposite was successfully synthesized^49^.

As known, overloading water in polymeric membranes causes swelling and rupturing membranes. The application of SA limited by its intrinsic weakness, such as low mechanical strength and high water-solubility, which could be conspicuous by transforming from SA to CSA. In addition to Ca^2+^, GOns could cross-link alginate chains together. Moreover, GOns could participate in the cross-linking of PA chains. Therefore, GOns can control water uptake, which makes lower swelling degrees. When the PC_1.5_P sample (with water uptake = 65%) was composed with GOns and PA layer, it ended with the generation of the optimized-nanocomposite sample of PC_1.5_PP that its water uptake value increased to 100%. It could originate from the hygroscopic nature of GOns that generate nano-channels for water retention^50,51^.

### The pore size distribution

In the dry state of samples that depicted in Fig. 2, the porosity of samples measured with the help of ImageJ software. As known, the tiny pores or the free-pore surfaces act as strong barriers to the fast transportation of the-contaminated fluid. According to the obtained results in Figs. S5–S8, the size and number of pores got a downward trend by constructing a new layer with different SA concentration, indicating that the accessible space for penetrating water molecules and salt ions is limited. Therefore, the achieved histograms showed that the average pore size dropped, signifying that the preparation of middle layer effectively decreased the pore size of PVDF nano-fibrous. Fortunately, the optimum sample of the GOns-integrated PC_1.5_PP had not shown any pore in its top layer, suggesting that the interfacial polymerization of PA completely filled the pores of support layer (Fig. S9). In addition, GOns effectively established the cross-linked state between polymeric chains. These could result in the feasible desalination performances than the other samples. Overall, these predictions had completely in accordance with the revealed observations^52–54^.

### An overview in the membrane desalination literatures

Seas and oceans are the primary sources of freshwater supply, and Reverse Osmosis (RO) is a principal technology for reaching this goal; meanwhile, RO needs to protect against destructive pollutants that usually cause fouling and performances loss^55^. The most outstanding system to attain this purpose is NF^56^. Therefore, in the scaling-up view, Kaya et al. focused on the two-step desalination process, where the commercial grade of NF module as a pre-treatment stage joined to a RO module for seawater desalination under 30–50 bar pressure. The results indicated that the combination of these membranes increased the quality of the permeated water^57^. In another piece of literature, Wolf et al. executed an Ultra-filtration unit to ensure reliable and stable operation of the RO system. Therefore, the pre-treatment process seems necessary for the stable and more suitable operation of the RO system^58^. Nevertheless, Li et al. and Zhang et al. discussed that the NF membranes suffer from the trade-off between SR and PWF, and tuning between surface charge and pore size distribution by coupling GOns could effectively enhance the desalination abilities^59,60^. Moreover, Park et al. demonstrated that the excellent stiffness of GOns could donate to the membrane by chemical cross-linking between Ca^2+^ ion and the oxygen-based functional group of GOns, suggesting that Ca^2+^ can act as a bridge between polymeric chains and GOns for sharing their best properties^61^. From another aspect and the closest work to our research, Chi et al. showed that a ternary layer of TFC membrane could end to the best desalination performances, when the support layer had a nano-fibrous structure^62^. Therefore, Liu et al. showed that the SR and PWF performances of TFC membrane universally improved under six bar, when the PA TFL produce by interfacial polymerization^63^. In addition, Jiang et al. revealed that preparing ultra-thin PA as the top-layer of TFC NF membrane enables scale membrane manufacturing that is not feasible as previously reported^64^. Rajesh and Bose reported that the top layer of PA could have the highest ion selectivity, when the top layer of the membrane was integrated with 12 bilayers of GOns^65^. Overall, it tried to prepare the GOns-integrated TFC NF membrane that can exhibit good performances considering these previous reports.

## Conclusion

In this work, the trinary membrane of PC_1.5_PP is fabricated by Step-by-Step processes of electrospinning, electrospraying@electrospinning, and interfacial polymerization for constructing the bottom, middle, and top layers. In addition, a green cross-linking agent used for controlling the swelling state of SA, establishing higher wettability, and enhancing PWF. The primary aim of preparing the PC_1.5_PP membrane was to achieve high SR and pleasant PWF, and the Taguchi method applied to optimize GOns percentage in each layer. Therefore, the plausible performances revealed when the layers of top, middle, and bottom had 1, 0.1, and 0.1 wt.% of GOns, respectively. In the optimum sample of PC_1.5_PP that contains the proper amounts of GOns, the SR and PWF achieved NaCl < MgCl_2_ < MgSO_4_ and 20.36 L/m^2^.h, respectively. Thus, it is noteworthy that all of these attempts can effectively be a promising potential for industrial applications of GOns-composited PC_1.5_PP membrane, as one of the emerging TFC NF membranes, when high PWF and satisfactory SR are essential.

## Supplementary Information

Supplementary Information.

## Data Availability

All data generated or analyzed during this study are included in this published article and its supplementary information file.
