# Supplementary material for: Integrating graphene oxide into layers of PVDF/PVDF@cross-linked sodium alginate/polyamide membrane for efficiently enhancing desalination performances
Source: Sci Rep. 2022 Oct 7;12:16908. doi: 10.1038/s41598-022-21316-y (PMC9546892; doi:10.1038/s41598-022-21316-y)
Supplement: Supplementary file 1 — Supplementary Information. [file 41598_2022_21316_MOESM1_ESM.docx]

**Supplementary data**

**Integrating Graphene Oxide into layers of PVDF//PVDF@Cross-linked Sodium Alginate//Polyamide membrane for efficiently enhancing desalination performances**

Additional Supporting Data

**Tables section:**

**Table S1.** The operational condition for electrospinning PVDF or electrospraying SA solutions.

**Table S2.** Introduced parameters and their levels to QUALITEK-4 software.

**Table S3.** The extracted experimental layout using the designed L9 orthogonal array.

**Table S4.** Analysis variance based on OEC results.

Figures section:

**Fig. S1.** The creation of egg-box state of CSA in middle layer of PC_1.5_PP membrane.

**Fig. S2.** The relation between Ca^2+^ concentration and PWF in PC_x_P samples.

**Fig. S3.** The SEM images of the aggregations of GOns in (a) middle layer and, (b) top layer of PC_1.5_PP sample.

**Fig. S4.** The FTIR spectra of samples.

**Fig. S5.** (a) The scanned surface of PVDF nanofibrous sample in Fig. 2(a) by ImageJ software, and (b) the histogram of the pore size distribution.

**Fig. S6.** (a) The scanned surface of PS_0.25_P sample in Fig. 2(b) by ImageJ software, and (b) the histogram of the pore size distribution.

**Fig. S7.** (a) The scanned surface of PS_1.5_P sample in Fig. 2(c) by ImageJ software, and (b) the histogram of the pore size distribution.

**Fig. S8.** (a) The scanned surface of PS_3_P sample in Fig. 2(d) by ImageJ software, and (b) the histogram of the pore size distribution.

**Fig. S9.** The SEM image of the GOns-integrated PC_1.5_PP sample.

**Table S1.** The operational condition for electrospinning PVDF or electrospraying SA solutions.

| **Applied voltages (kV)** | **Flow rate (mL.h^-1^)** | **Needle to collector distance (cm)** | **Rotating speed (rpm)** | **Ambient temperature (°C)** |
| --- | --- | --- | --- | --- |
| 22 | 1 | 15 | 300 | 27-29 |

**Table S2.** Introduced parameters and their levels to QUALITEK-4 software.

| Parameters | Levels | | |
| --- | --- | --- | --- |
|  | 1 | 2 | 3 |
| GOns %wt in top layer (G1) | 0.1 | 0.5 | 1 |
| GOns %wt in middle layer (G2) | 0.1 | 0.5 | 1 |
| GOns %wt in bottom layer (G3) | 0.1 | 0.5 | 1 |

**Table S3.** The extracted experimental layout using the designed L9 orthogonal array.

| Experimental number | Amount of levels | | |
| --- | --- | --- | --- |
|  | G1 | G2 | G3 |
| S1 | 0.1 | 0.1 | 0.1 |
| S2 | 0.5 | 0.5 | 0.1 |
| S3 | 1 | 1 | 0.1 |
| S4 | 0.5 | 0.1 | 0.1 |
| S5 | 1 | 0.5 | 0.5 |
| S6 | 0.1 | 1 | 0.5 |
| S7 | 1 | 0.1 | 1 |
| S8 | 0.1 | 0.5 | 1 |
| S9 | 0.5 | 1 | 1 |

**Table S4.** Analysis variance based on OEC results.

| **Response** | **Relative weight (%)** | | **Factors** | | **DOF** | **Sum of square** | **Variance** | **Portion (%)** |
| --- | --- | --- | --- | --- | --- | --- | --- | --- |
| PWF | | 50 | | G1 | 2 | 738.88 | 369.44 | 57.50 |
|  |  |  |  | G2 | 2 | 166.22 | 80.11 | 10.59 |
| SR | | 50 | | G3 | 2 | 304.88 | 152.44 | 22.32 |
|  | |  | | Error | 2 | 14.77 | 14.77 | 9.58 |


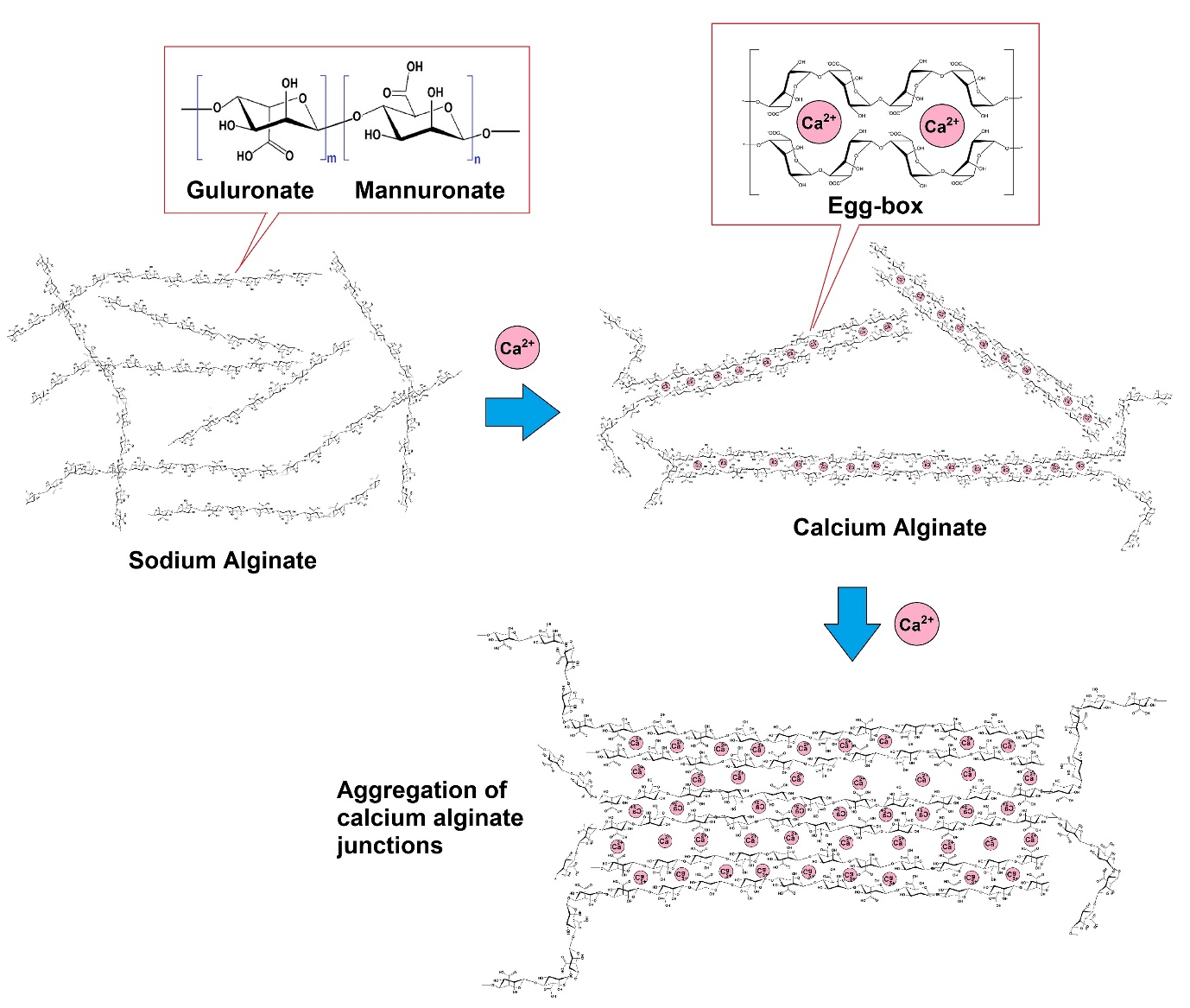


**Fig. S1.** The creation of egg-box state of CSA in middle layer of PC_1.5_PP membrane.


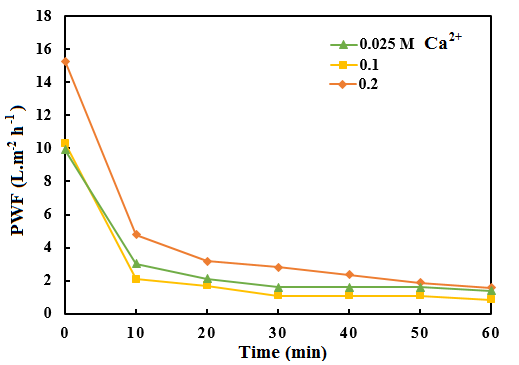


**Fig. S2.** The relation between Ca^2+^ concentration and PWF in PC_x_P samples.


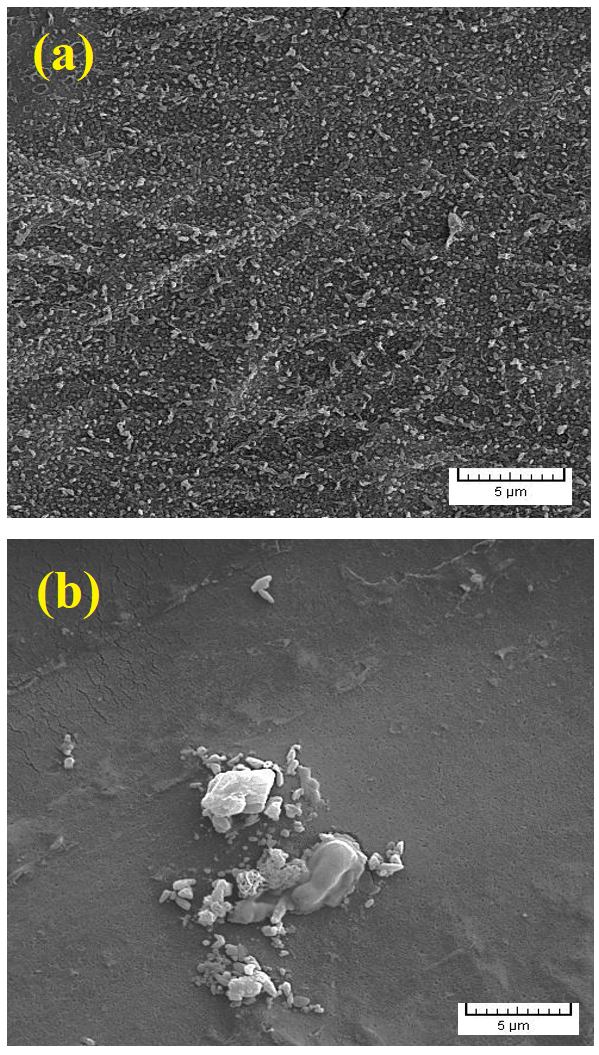


**Fig. S3.** The SEM images of the aggregations of GOns in (a) middle layer and, (b) top layer of PC_1.5_PP sample.


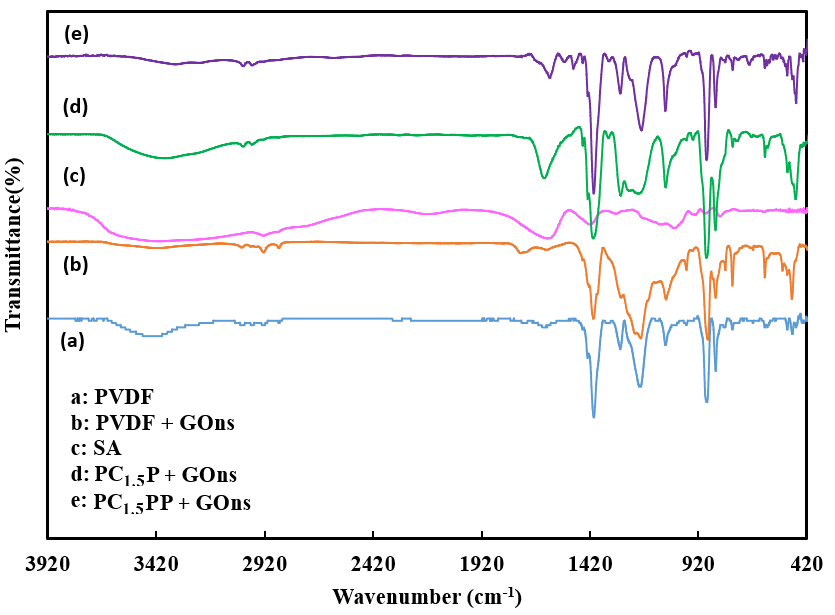


**Fig. S4.** The FTIR spectra of samples.


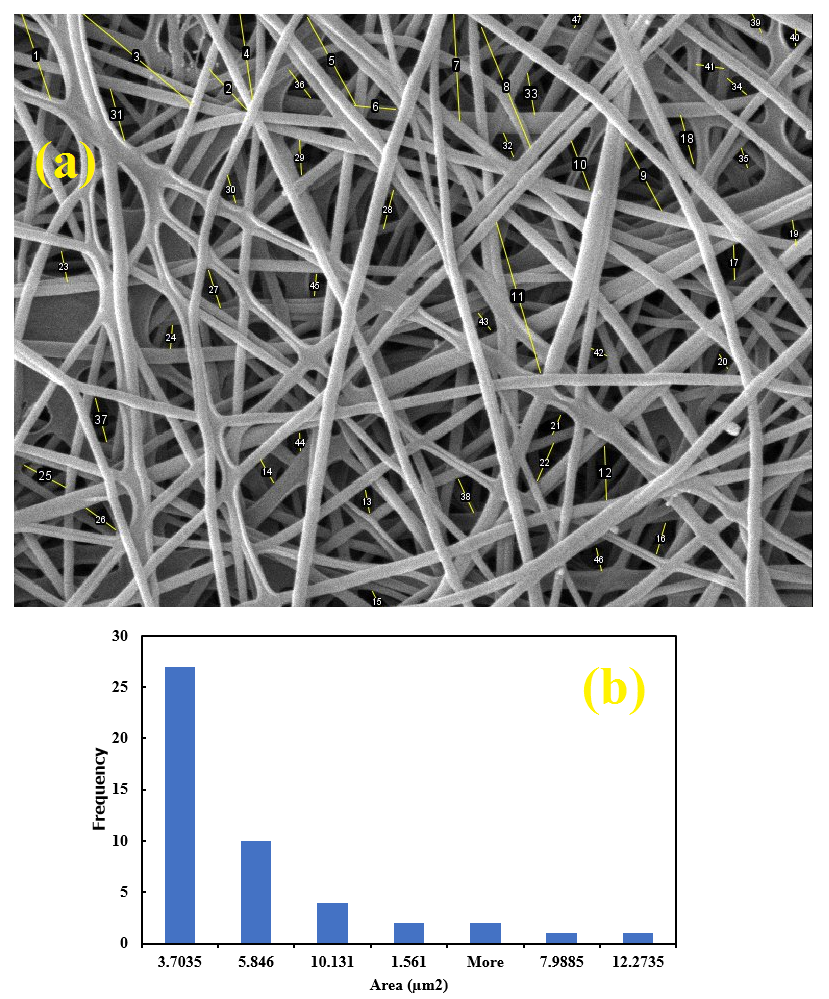


**Fig. S5.** (a) The scanned surface of PVDF nanofibrous sample in Fig. 2(a) by ImageJ software, and (b) the histogram of the pore size distribution.


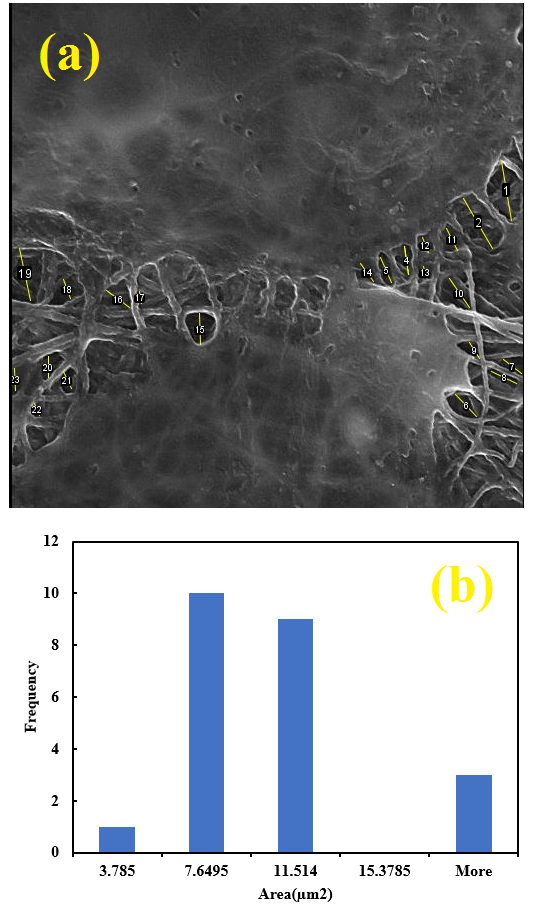


**Fig. S6.** (a) The scanned surface of PS_0.25_P sample in Fig. 2(b) by ImageJ software, and (b) the histogram of the pore size distribution.


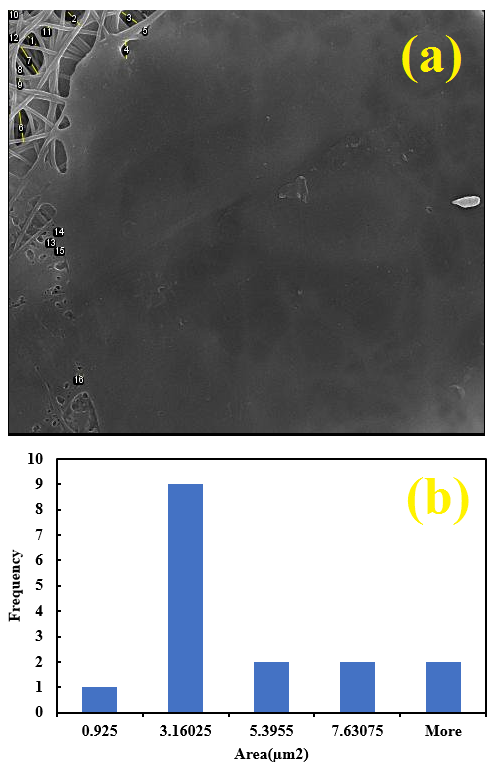


**Fig. S7.** (a) The scanned surface of PS_1.5_P sample in Fig. 2(c) by ImageJ software, and (b) the histogram of the pore size distribution.


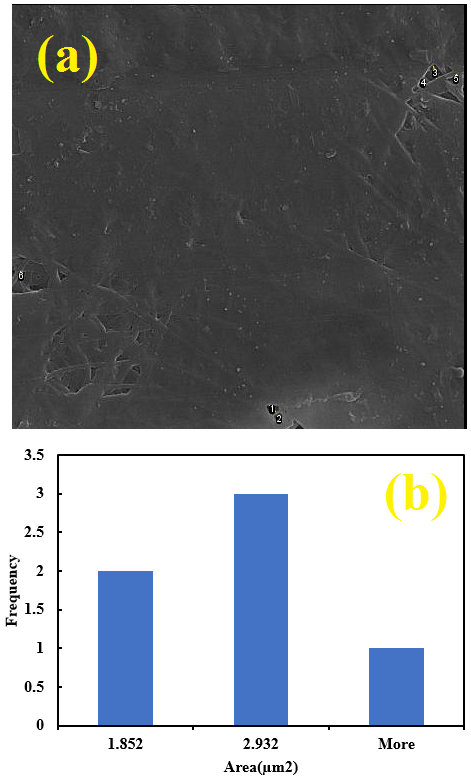


**Fig. S8.** (a) The scanned surface of PS_3_P sample in Fig. 2(d) by ImageJ software, and (b) the histogram of the pore size distribution.


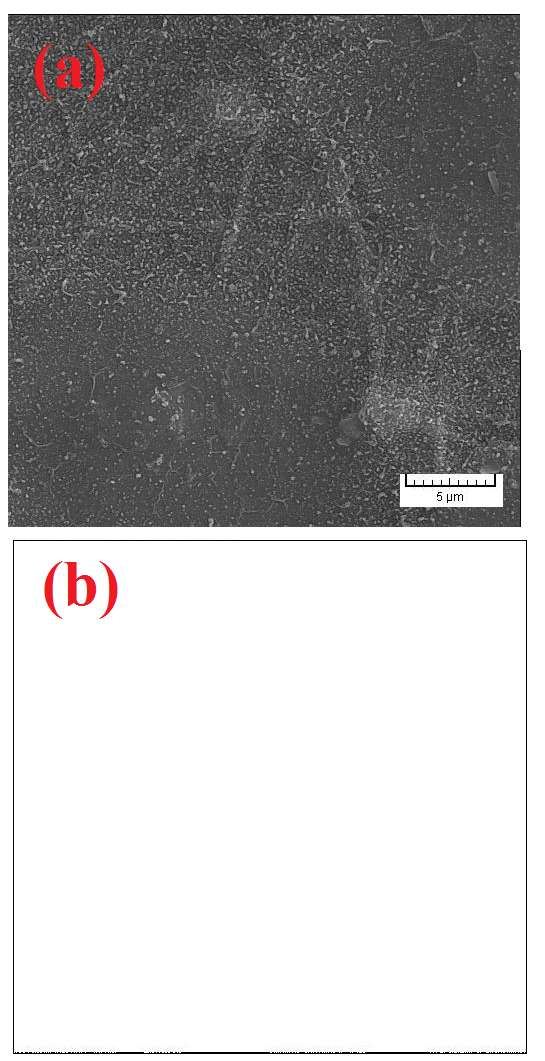


**Fig. S9.** The SEM image of the GOns-integrated PC_1.5_PP sample
